# Body surface potential mapping of ventricular depolarization and repolarization in phospholamban and plakophilin-2 cardiomyopathy

**DOI:** 10.1016/j.hroo.2025.09.027

**Published:** 2025-10-17

**Authors:** Iris van der Schaaf, Manon Kloosterman, Machteld J. Boonstra, Rob W. Roudijk, Anneline S.J.M. te Riele, Peter M. van Dam, Peter Loh

**Affiliations:** 1 Department of Cardiology, University Medical Center Utrecht, Utrecht, The Netherlands; 2 Department of Cardiology, Amsterdam Cardiovascular Sciences, Amsterdam University Medical Center, University of Amsterdam, Amsterdam, The Netherlands; 3 Department of Cardiology, Radboud University Medical Center Nijmegen, Nijmegen, The Netherlands; 4 Jagiellonian University Medical College, Center for Digital Medicine and Robotics, Krakow, Poland

**Keywords:** Electrocardiogram, Cardiac depolarization and repolarization, R-, S-, and T-wave amplitudes, Arrhythmogenic cardiomyopathy, Body surface potential mapping

## Abstract

**Background:**

Pathogenic variants in plakophilin*-*2 (*PKP2*) and phospholamban (*PLN*) are associated with arrhythmogenic cardiomyopathy. Early disease detection is important to prevent adverse events. Body surface potential mapping (BSPM) may detect local electrical abnormalities earlier than the 12-lead electrocardiogram.

**Objective:**

This study aimed to determine abnormalities in R-, S-, and T-wave amplitudes in *PKP2-* and *PLN-*pathogenic variant carriers using BSPM.

**Methods:**

67 lead BSPM was performed in controls and *PKP2* and *PLN* carriers. R-, S-, and T-wave amplitudes across all leads in controls were used as reference. Amplitudes of carriers exceeding these ranges were considered abnormal and assessed across disease stages (presymptomatic, electrical, and structural, as done previously). Follow-up BSPM (≥2 years) was performed in a subset of carriers.

**Results:**

152 subjects (40 [27;54] years; 51% women) (40 controls and 112 carriers [53 *PKP2* and 59 *PLN*]) were included. Amplitude abnormalities were most frequent in structural disease, predominantly in T waves (*PKP2* 20 [10;29]; *PLN* 25 [22;30] leads). Abnormalities in electrical disease were more prevalent in *PLN* carriers than *PKP2* carriers (R wave 4 [1;7] vs 13 [8;16] leads, *P* = .002; S wave 2 [1;3] vs 4 [3;12] leads, *P* < .001; T wave 1 [0;3] vs 20 [16;28] leads, *P* < .001). Presymptomatic carriers typically had abnormalities outside the 12-lead configuration. As the disease progressed, abnormalities became more frequent and extended toward V1–V6. Follow-up BSPM (23 *PKP2* and 16 *PLN*) showed consistency in locations of abnormalities with increased frequency (maximal increase 31%).

**Conclusion:**

BSPM detected abnormal amplitudes within and beyond the 12-lead electrocardiogram, even in presymptomatic carriers. Follow-up BSPM suggests that these abnormalities are associated with disease progression, highlighting the potential benefit of BSPM in early disease detection.

**Key Findings**

▪ Abnormal R-, S-, and T-wave amplitudes were detected within and beyond the 12-lead configuration in pathogenic variant carriers associated with arrhythmogenic cardiomyopathy.
▪ Abnormalities were also detectable in presymptomatic variant carriers, typically occurring outside standard precordial lead positions.
▪ Findings of follow-up body surface potential mapping suggest that these abnormalities are associated with disease progression.

## Introduction

The standard 12-lead electrocardiogram (ECG) plays an important role in detecting both the presence and progression of disease in individuals at risk of developing arrhythmogenic cardiomyopathy (ACM). Prolonged terminal activation duration and T-wave inversions are part of the 2010 Task Force Criteria (TFC) used to diagnose arrhythmogenic right ventricular cardiomyopathy (ARVC), the best characterized subform of ACM.^1^ Although the TFC are effective in identifying the classic right ventricular (RV) phenotype of the disease, particularly in desmosomal variants such as plakophilin-2 (*PKP2*), there is a broader spectrum of ACM, which also includes left-sided nondesmosomal variants like phospholamban (*PLN*), that is not covered by the TFC.^2^, ^3^, ^4^ These variants often show different ECG characteristics; for example, *PLN*-related disease is typically characterized by low-voltage ECG signals, which are not included in the TFC.^5^

Given the variability in disease expression across different genetic subtypes,^4^^,^^6^ it is also critical to consider the spatial distribution of myocardial involvement when assessing ECG abnormalities, given that the standard 12-lead ECG precordial positions do not reflect all myocardial regions. To detect the depolarization and repolarization abnormalities in more diverse ACM phenotypes, it is suggested to use 67-lead body surface potential mapping (BSPM). BSPM is a noninvasive technique that offers a more comprehensive assessment of cardiac electrical activity than the standard 12-lead ECG. BSPM has demonstrated utility in various cardiac conditions,^7^, ^8^, ^9^ among which is *PKP2* cardiomyopathy.^10^, ^11^, ^12^

In this study, we aimed to investigate 67-lead BSPM in carriers of *PKP2* and *PLN* genetic variants and describe the spatial distribution of ECG abnormalities across these different genetic subtypes. We hypothesize that BSPM is better able to detect de- and repolarization abnormalities than the standard 12-lead ECG, especially in early disease stages.

## Methods

### Study population

Subjects who were ≥18 years old and who had a class V pathogenic variant in *PKP2* or *PLN* as per American College of Medical Genetics and Genomics criteria^13^ were included. Control subjects were included if they were ≥18 years old and were (1) healthy volunteers, (2) athletes, or (3) referred for cardiac magnetic resonance imaging (CMR) or computed tomography (CT) and did not fulfill any of the following exclusion criteria: structural abnormalities as determined per echocardiography/CMR/CT, use of antiarrhythmic medication other than beta-blockers, previous ablation of ventricular arrhythmias, presence of (nonsustained) ventricular arrhythmias, or premature ventricular contractions (PVCs) in bigeminy.

The study was performed according to the Declaration of Helsinki, and the study protocols were approved by the medical ethics review board of the University Medical Center Utrecht (#17-907). All study subjects provided oral and written informed consent before the study.

### Data collection

**Figure 1:**
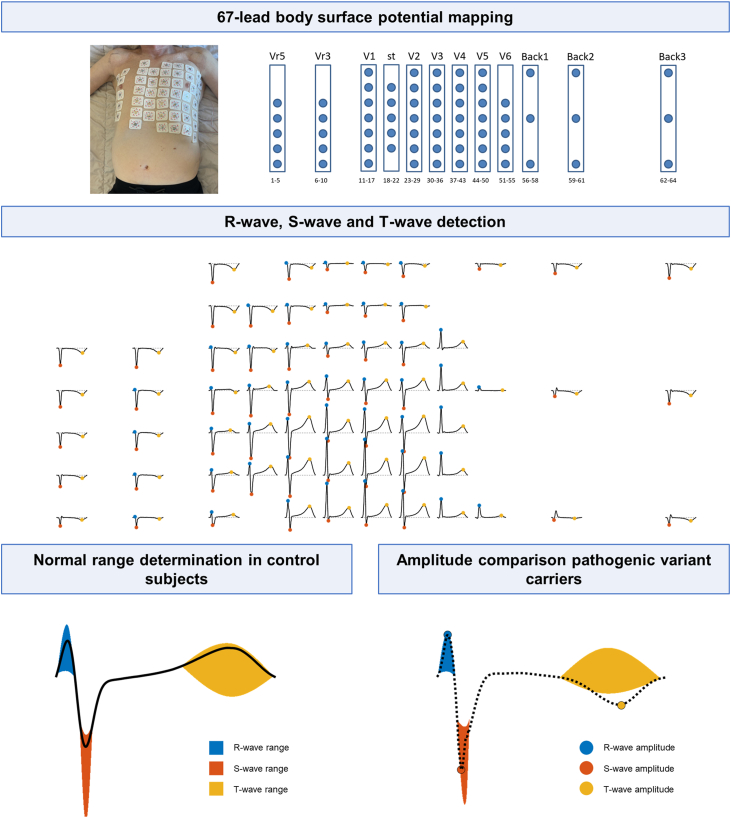
Methodological workflow of the study: (1) setup of the torso electrodes from the 67-lead body surface potential mapping (BSPM) system; (2) R-, S-, and T-wave detection in a 67-lead BSPM; (3) normal range determination based on all 40 control subjects; and (4) abnormal amplitude detection of a T wave in plakophlin-2 pathogenic variant carrier.

Sixty-seven-lead BSPM was performed (Biosemi, Amsterdam, The Netherlands) with 9 electrodes on the back, 55 chest electrodes, and 3 extremity electrodes positioned in the standard configuration (Figure 1). The extremity electrodes were used to compute Wilson’s central terminal, which was used as a reference for the chest and back electrodes. The electrodes were positioned using 12 vertical strips with 4 cm between the chest electrodes and 9 cm between the back electrodes. The position of the electrodes on the torso was recorded using a 3-dimensional camera (Intel, Santa Clara, CA) and a dedicated software (PeacsCamera, Version 0.0.2.6769, Peacs Investments BV).

BSPM was continuously recorded with a 2048 Hz sample frequency. A baseline BSPM was recorded for approximately 5 minutes in the supine position. A follow-up BSPM was recorded in a subset of the pathogenic variant carriers, with a minimum follow-up duration of 2 years. The 3-dimensional camera recordings from the initial BSPM were used as a reference for the placement of electrodes during follow-up BSPM.

### Data processing

BSPM signals were analyzed using MATLAB (version R2023B, MathWorks, Natick, MA). Signals were down sampled from 2048 Hz to 1000 Hz using the resample function in MATLAB and separately filtered using a Butterworth filter (high-pass: fc = 0.1 Hz).

A median beat was computed for every lead based on all beats from 15 seconds of recording. Median beats were created by aligning individual complexes on the R peak, as detected by a Pan-Tompkins-based algorithm.^14^ The root mean square signal, calculated with all 67 median beats, was used to manually annotate the global QRS onset and QRS end. T end was detected using a previously described integration operation method.^15^ All individual beats were baseline corrected between QRS onset and T end by linear interpolation before calculating the median. All annotations and median beats were checked manually.

### Data analysis

The R-, S-, and T-wave amplitudes across all 67 leads were determined for each subject (Figure 1). R- and S-wave peaks were automatically detected and considered valid if they met duration (≈12 ms) and amplitude criteria (≥30 μV for at least 6 ms) as previously established.^16^ R and S waves that did not meet the criteria were excluded from analysis. T-wave peaks were detected as the maximum or minimum deflection between QRS end of +70 ms and T end.

The R-, S-, and T-wave amplitudes from the control group served as a normal reference (Figure 1). To define this normal reference, the minimum and maximum R-, S-, and T-wave amplitudes were determined for each lead, and the upper and lower 2.5 percentiles were excluded to eliminate outliers. The amplitudes of the pathogenic variant carriers were then evaluated against this normal reference. Any amplitude falling outside the defined normal range for a given lead was classified as abnormal.

**Figure 2:**
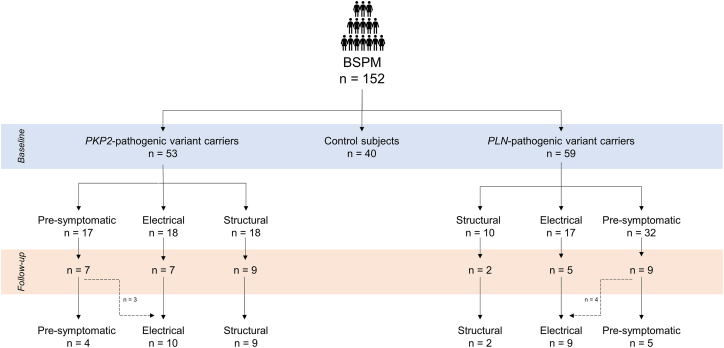
Flowchart of the study population. BSPM = body surface potential mapping; PKP2 = plakophilin-2; PLN = phospholamban.

The described analysis was performed on a group level but also stratified by disease stage and pathogenic variant, as displayed in Figure 2. For *PKP2*-pathogenic variant carriers, the disease stages were based on the 2010 TFC^1^^,^^17^: (1) presymptomatic, no TFC present other than the presence of a pathogenic variant; (2) electrical stage, presence of depolarization and/or repolarization TFC and/or presence of arrhythmia TFC or history of any ventricular arrhythmias without presence of structural TFC; and (3) structural disease stage, TFC fulfillment of structural abnormalities regardless of presence of other TFC. Given that TFC are focused on RV abnormalities and known to be insufficient in *PLN*-pathogenic variant carriers, different, previously established criteria^18^ were used to determine the disease stage in this group. *PLN* carriers were categorized as (1) presymptomatic (preserved left ventricular ejection fraction [LVEF] [≥45%], a PVC count of <500 per 24 hours, and no history of ventricular arrhythmias), (2) electrical (history of ventricular arrhythmias and/or a PVC count of ≥500 per 24 hours but who had an LVEF of ≥45%), and (3) structural (an LVEF of <45% regardless of the presence of PVCs or ventricular arrhythmias). The number of abnormal R-, S-, and T-wave amplitudes was also determined in pathogenic variant carriers with and without ventricular arrhythmias, RV and left ventricular (LV) dyskinesia, and T-wave inversions.

### Data visualization and statistics

The frequency of abnormality in R-, S-, and T-wave amplitudes per lead was plotted on a general torso model with a standard 67-lead configuration, where a higher color saturation indicated a higher frequency of abnormal R-, S-, and T-wave amplitudes, where 100% indicates an abnormal lead in 100% of the subjects in that group (per genetic variant, per disease stage) and in those with and without late gadolinium enhancement (LGE).

To compare between BSPM and the precordial leads of the 12-lead ECG, the lead with the highest frequency of abnormal R-, S-, and T-wave amplitudes was identified within leads V1–V6 and within the remaining 67 leads (excluding V1–V6).

Statistical analysis was performed using R (version 2024.06.05, R Foundation for Statistical Computing, Vienna, Austria). Categorical data were presented as numbers (percentages). Continuous data were presented as median [Q1;Q3]. Unpaired data were tested for statistical significance using an independent sample *t* test or Mann-Whitney U test, as appropriate. Paired data were tested for statistical significance using a sample *t* test or Wilcoxon signed-rank test, as appropriate. Spearman’s rank correlation coefficient was determined between LVEF/RV ejection fraction (RVEF) and the number of abnormal R-, S-, and T-wave amplitudes per genetic variant.

*P* < .05 was considered significant. However, owing to multiple comparisons in the analysis of baseline BSPM, *P* values were adjusted using Bonferroni correction. A *P* value was considered statistically significant if *P* < .004 in this subanalysis.

## Results

### Baseline characteristics

In total, 152 subjects were included (age 40 [27;54] years; 51% women), of whom 40 were control subjects and 112 pathogenic variant carriers. Baseline characteristics are presented in Table 1. 53 subjects were carriers of a *PKP2*-pathogenic variant, and 59 had a *PLN*-pathogenic variant. Of the pathogenic variant carriers, 49 (44%) were in a presymptomatic stage of disease, 35 (31%) in an electrical disease stage, and 28 (25%) in a structural disease stage (Figure 2).

**Table 1:** Baseline characteristics of all subjects and stratified by genetic variant

Baseline characteristics	All (n = 152)	Controls (n = 40)	*PKP2* (n = 53)	*PLN* (n = 59)
Age (y)	40 [27;54]	36 [26;51]	33 (25, 54)	43 (29, 60)
Sex, female (n)	77 (51)	15 (39)	24 (45)	38 (64)
BMI (kg/m^2^)	23.7 [21.6;25.6]	22.8 [21.0;25.1]	24.1 [22.0;27.1]	23.4 [21.3;25.0]
Antiarrhythmic medication (n)	31 (25)	4 (10)	6 (11)	21 (36)
HF medication (n)	29 (24)	0 (0)	4 (8)	25 (42)
Heart rate baseline BSPM (bpm)	62 [56;70]	59 [55;66]	62 [56;70]	64 [58;71]
TFC fulfillment				
TFC of ≤2 (n)	92 (65)	40 (100)	18 (34)	44 (75)
TFC of 3 (n)	17 (12)	0 (0)	12 (23)	5 (8.5)
TFC of ≥4 (n)	33 (23)	0 (0)	23 (43)	10 (17)
Depolarization TFC fulfillment				
Prolonged TAD (n)	15 (10)	0 (0)	12 (23)	3 (5)
Repolarization TFC fulfillment				
T-wave inversion, leads V1 and V2 (n)	6 (4)	0 (0)	3 (6)	3 (5)
-wave inversion, leads V1–V3 (n)	10 (7)	0 (0)	10 (19)	0 (0)
T-wave inversion, leads V4, V5, or V6 (n)	13 (9)	0 (0)	2 (4)	11 (19)
T-wave inversion, leads V1–V6 (n)	3 (2)	0 (0)	1 (2)	2 (3)
T-wave inversion with RBBB, leads V1–V4 (n)	4 (3)	0 (0)	0 (0)	4 (7)
Arrhythmia TFC fulfillment				
>500 VES per 24 h (n)	37 (24)	4 (10)	18 (34)	15 (25)
(ns)VT of LBBB morphology with superior axis (n)	11 (7)	0 (0)	8 (15)	3 (5)
(ns)VT of RV outflow configuration, LBB morphology with inferior axis or of unknown axis (n)	5 (3)	0 (0)	3 (6)	2 (3)
(ns)VT of unknown morphology and axis (n)	22 (14)	0 (0)	11 (21)	11 (19)
CMR characteristics				
CMR presence of LV dyskinesia/akinesia (n)	10 (7)	0 (0)	1 (2)	9 (15)
CMR presence of RV dyskinesia/akinesia (n)	30 (20)	0 (0)	15 (28)	15 (25)
LVEDV/BSA (mL/m^2^)	93 [81;101]	88 [82;94]	92 [80;99]	94 [83;104]
RVEDV/BSA (mL/m^2^)	95 [83;111]	89 [83;103]	97 [81;118]	93 [84;106]
LVEF ()	55 [51;59]	56 [55;59]	56 [53;60]	54 [49;57]
RVEF ()	53 [47;58]	54 [53;57]	53 [47;59]	52 [46;57]
LV LGE (n)	37 (24)	0 (0)	9 (17)	28 (47)
RV LGE (n)	14 (9)	0 (0)	8 (15)	6 (10)
Echocardiographic characteristics				
Echo presence of LV dyskinesia/akinesia (n)	10 (7)	0 (0)	2 (4)	8 (14)
Echo presence of RV dyskinesia/akinesia (n)	17 (11)	0 (0)	12 (23)	5 (8)
Echo LVEF (%)	55 [51;59]	62 [60;63]	56 [54;60]	55 [46;57]
RVOT PLAX (mm)	31.0 [28.0;36.0]	35.5 [35.0;36.0]	31.0 [28.0;35.0]	30.5 [28.0;36.0]
RVOT PSAX (mm)	33.0 [29.0;37.0]	36.5 [36.0;37.0]	33.0 [29.5;38.5]	31.0 [29.0;34.0]

Values are presented as number (%) or median [Q1;Q3].

bpm = beats/min; BMI = body mass index; BSA = body surface area; BSPM = body surface potential mapping; CMR = cardiac magnetic resonance imaging; EDV = end diastolic volume; EF = ejection fraction; HF = heart failure; LBBB = left bundle branch block; LGE = late gadolinium enhancement; LV = left ventricle; (ns)VT = (nonsustained) ventricular tachycardia; PKP2 = plakophilin-2; PLAX = parasternal long axis; PLN = phospholamban; PSAX = parasternal short axis; RBBB = right bundle branch block; RV = right ventricle; RVOT = right ventricular outflow tract; TAD = terminal activation duration; TFC = Task Force Criteria.

The median heart rate was 62 beats/min (bpm) [56;70] and was similar among controls (59 [55;66] bpm), *PKP2* carriers (62 [56;70] bpm), and *PLN* carriers (64 [58;71] bpm).

Overall, 23 *PKP2* carriers (43%) had a definite ARVC diagnosis according to the TFC compared with 10 of the *PLN* carriers (17%). Left-sided T-wave inversions were more commonly observed in *PLN* carriers (19%), and right-sided T-wave inversions were more common in *PKP2* carriers (19%). (Non-)sustained ventricular arrhythmias were present in 24% of all pathogenic variant carriers. CMR showed generally preserved biventricular ejection fractions. LV LGE and dys-/akinesia were more prevalent in *PLN* carriers than in *PKP2* carriers (47% vs 17% and 15% vs 2%, respectively), whereas RV late enhancement and dys-/akinesia were more prevalent in *PKP2* carriers than in *PLN* carriers (15% vs 10% and 28% vs 25%, respectively).

### Baseline body surface potential map

#### *PKP2* carriers

Among all *PKP2* carriers, the R-, S-, and T-wave amplitudes were found to be abnormal in 5 [2;8], 4 [2;6], and 3 leads [0;16], respectively (Table 2). A clear trend was observed where the number of leads with abnormal amplitudes increased per disease stage; the lowest number of abnormal leads was observed in the presymptomatic stage, whereas the highest number of abnormal leads was observed in the structural stage (R wave 2 [1;5] vs 9 [5;16] leads, *P* = .004; S wave 4 [1;6] vs 7 [5;12] leads, *P* = .022; T wave 0 [0;4] vs 20 [10;29] leads, *P* < .001). Notably, T-wave abnormalities were rare in both presymptomatic and electrical disease stages (0 [0;4] vs 1 [0;3] leads; *P* = .958), whereas a sudden increase was observed in the structural stage (20 [10;29] leads) compared with presymptomatic carriers (*P* < .001) and carriers with electrical disease (*P* < .001).

**Table 2:** Number of abnormal leads for *PKP2*-pathogenic variant carriers and PLN-pathogenic variant carriers during baseline body surface potential mapping

Baseline BSPM	All	Presymptomatic	Electrical	Structural	*P* value, presymptomatic vs electrical	*P* value, presymptomatic vs structural	*P* value, electrical vs structural
*PKP2*	n = 53	n = 17	n = 18	n = 18			
Abnormal R wave (n)	5 [2;8]	2 [1;5]	4 [1;7]	9 [5;16]	.378	.004	.016
Abnormal S wave (n)	4 [2;6]	4 [1;6]	2 [1;3]	7 [5;12]	.077	.022	<.001∗
Abnormal T wave (n)	3 [0;16]	0 [0;4]	1 [0;3]	20 [10;29]	.958	<.001∗	<.001∗
*PLN*	n = 59	n = 32	n = 17	n = 10			
Abnormal R wave (n)	10 [4;17]	5 [3;11]	13 [8;16]	18 [15;22]	.031	<.001∗	.062
Abnormal S wave (n)	4 [2;10]	3 [0;8]	4 [3;12]	11 [7;16]	.050	.001∗	.041
Abnormal T wave (n)	16 [1;26]	1 [0;14]	20 [16;28]	25 [22;30]	.001∗	<.001∗	.118

Values are presented as median [Q1;Q3].

*BSPM* = body surface potential mapping; *PKP2* = plakophilin-2; *PLN* = phospholamban.

∗ A *P* value after Bonferroni correction was considered significant if *P* < .004.

**Figure 3:**
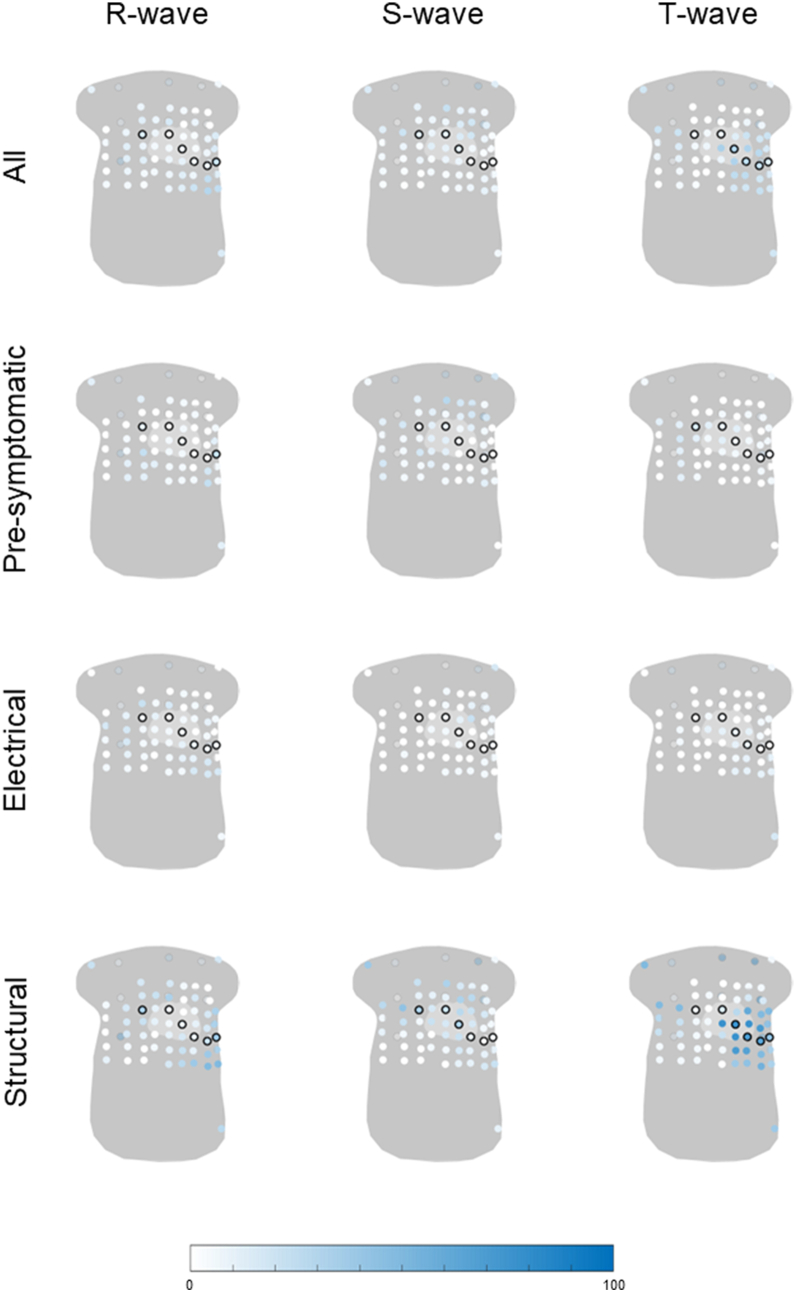
Percentage of abnormal R-, S-, or T-wave amplitude in all plakophilin-2 pathogenic variant carriers and plakophilin-2 pathogenic variant carriers in the disease stages: presymptomatic (n = 17), electrical (n = 18), or structural (n = 18). All 67 electrodes are visualized on a general torso model, and the frequency of abnormality is visualized from *white* (0%) to *blue* (100%), where a higher color saturation indicates a higher frequency of abnormal R-, S-, and T-wave amplitudes. The standard precordial electrodes V1–V6 and the back electrodes are circled in *black*.

The percentage of abnormal R-, S-, and T-wave amplitudes in all *PKP2*-pathogenic variant carriers is displayed in Figure 3. The R-wave amplitude was most frequently abnormal in leads located in the lower left side of the torso (lead with the highest frequency 30%). Abnormal S-wave amplitudes were predominantly observed on the back and in the upper side of the torso (lead with the highest frequency 30%). Abnormal T-wave amplitudes were distributed across the upper right side, the (lower) left side, and the back of the torso (lead with the highest frequency 34%). In presymptomatic carriers, abnormalities were seen in right-sided and back leads (leads with the highest frequencies, R wave 24%; S wave 35%; T wave 18%). For the R wave, abnormalities were also observed in the left lower leads. In the electrical stage, abnormalities were less frequent, with the R wave being most often affected in the right upper and left lower leads (lead with the highest frequency 22%). In the structural stage, the R wave was most frequently abnormal in the right upper and left lower leads (lead with the highest frequency 50%), whereas the S wave showed abnormalities in the (right) upper and precordial leads V1–V3 (lead with the highest frequency 44%). The T wave was frequently abnormal in structural disease, especially in (left) precordial leads V3–V6, left lower leads, and right upper leads (lead with the highest frequency 78%).

When assessing the frequency of abnormalities within and outside the standard precordial lead positions, the lead with the highest frequency of abnormal R-, S-, and T-wave amplitudes was often observed outside the standard ECG, particularly in carriers in the electrical disease stage (R wave [12-lead] 11%, BSPM 22%; S wave [12-lead] 6%, BSPM 22%; T wave [12-lead] 11%, BSPM 17%) (Table 3). Among carriers in the structural disease stage, higher frequencies of abnormal amplitudes were observed in BSPM leads for the R wave (12-lead 39%; BSPM 50%) and S wave (12-lead 33%; BSPM 44%). In the presymptomatic stage, only the S wave showed a higher frequency of abnormalities in BSPM leads (12-lead 12%; BSPM 35%), whereas, for the R and T waves, the highest percentage of abnormal amplitude was the same between BSPM and standard precordial leads (R wave 12%; T wave 18%).

**Table 3:** The highest observed frequency in R-, S-, and T-wave amplitudes within the precordial leads V1–V6 of the 12-lead ECG and the remaining leads of the BSPM

Highest observed frequency	12-lead	BSPM
*PKP2*		
Highest observed percentage of abnormal R waves within 1 lead		
Presymptomatic	24%	24%
Electrical	11%	22%
Structural	39%	50%
Highest observed percentage of abnormal S waves within 1 lead		
Presymptomatic	12%	35%
Electrical	6%	22%
Structural	33%	44%
Highest observed percentage of abnormal T waves within 1 lead		
Presymptomatic	18%	18%
Electrical	11%	17%
Structural	78%	78%
*PLN*		
Highest observed percentage of abnormal R waves within 1 lead		
Presymptomatic	31%	38%
Electrical	59%	76%
Structural	80%	90%
Highest observed percentage of abnormal S waves within 1 lead		
Presymptomatic	19%	44%
Electrical	35%	47%
Structural	50%	60%
Highest observed percentage of abnormal T waves within 1 lead		
Presymptomatic	25%	34%
Electrical	76%	82%
Structural	100%	100%

BSPM = body surface potential mapping; ECG = electrocardiogram; *PKP2* = plakophilin-2; *PLN* = phospholamban.

#### *PLN* carriers

Among all *PLN* carriers, R-, S-, and T-wave amplitudes were considered abnormal in 10 [4;17], 4 [2;10], and 16 leads [1;26], respectively (Table 2). R-, S-, and T-wave abnormalities were also observed in presymptomatic variant carriers, but a significantly higher amount of abnormalities were observed in those with structural disease (R wave 5 [3;11] vs 18 [15;22] leads, *P* < .001; S wave 3 [0;8] vs 11 [7;16] leads, *P* = .001; T wave 1 [0;14] vs 25 [22;30] leads, *P* < .001). A significant increase in T-wave abnormalities was also observed between presymptomatic variant carriers and those in the electrical disease stage (1 [0;14] vs 20 [16;28] leads; *P* = .001). Compared with *PKP2*-variant carriers in the electrical disease stage, *PLN* carriers in the electrical disease stage showed more R-, S-, and T-wave abnormalities (R wave 4 [1;7] vs 13 [8; 16] leads, *P* = .002; S wave 2 [1;3] vs 4 [3;12] leads, *P* < .001; T wave 1 [0;3] vs 20 [16;28] leads, *P* < .001).

**Figure 4:**
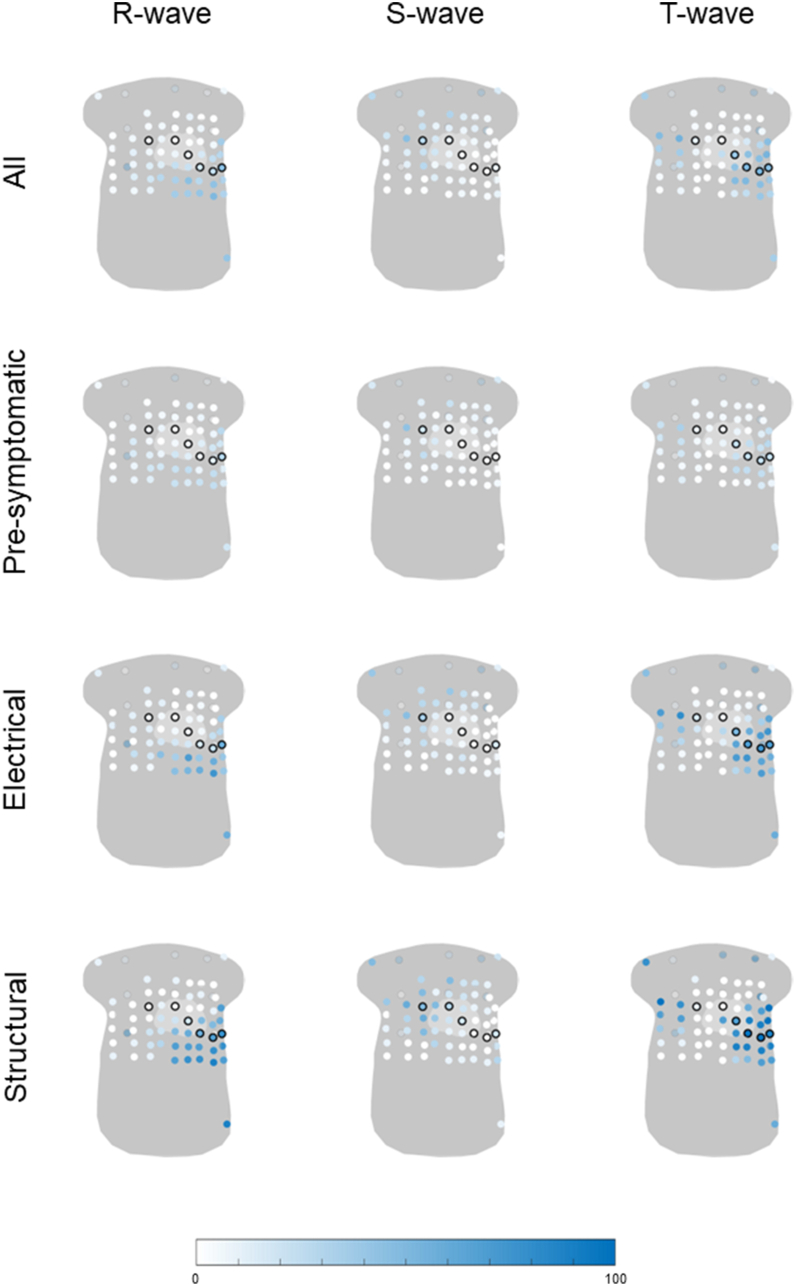
Percentage of abnormal R-, S-, or T-wave amplitude in all phospholamban pathogenic variant carriers and phospholamban pathogenic variant carriers in the disease stages: presymptomatic (n =32), electrical (n = 17), or structural (n = 10). All 67 electrodes are visualized on a general torso model, and the frequency of abnormality is visualized from *white* (0%) to *blue* (100%), where a higher color saturation indicates a higher frequency of abnormal R-, S-, and T-wave amplitudes. The standard precordial electrodes V1–V6 and the back electrodes are circled in *black*.

The percentage of abnormal R-, S-, and T-wave amplitudes per lead in *PLN*-pathogenic variant carriers is displayed in Figure 4. The R-wave amplitude was most frequently abnormal in the lower left and left axillary leads (lead with the highest frequency 51%). The S wave was most frequently abnormal in the upper right leads and back leads (lead with the highest frequency 47%), and the T wave was abnormal in the upper right, lower left, and back leads (lead with the highest frequency 56%). The regions in which abnormal leads were observed were largely consistent across disease stages; however, with disease progression, both the spatial extent of abnormalities and the frequency of abnormal leads increased (leads with the highest frequencies in the presymptomatic stage: R wave 38%, S wave 44%, T wave 34%; electrical stage: R wave 76%, S wave 47%, T wave 82%; structural stage: R wave 90%, S wave 60%, T wave 100%).

The highest frequencies of abnormal R-, S-, and T-wave amplitudes were consistently observed in leads outside the standard precordial positions (Table 3). An exception was the T wave in carriers in the structural disease stage, where identical frequencies were found within and outside the precordial leads (100%).

### LGE

**Figure 5:**
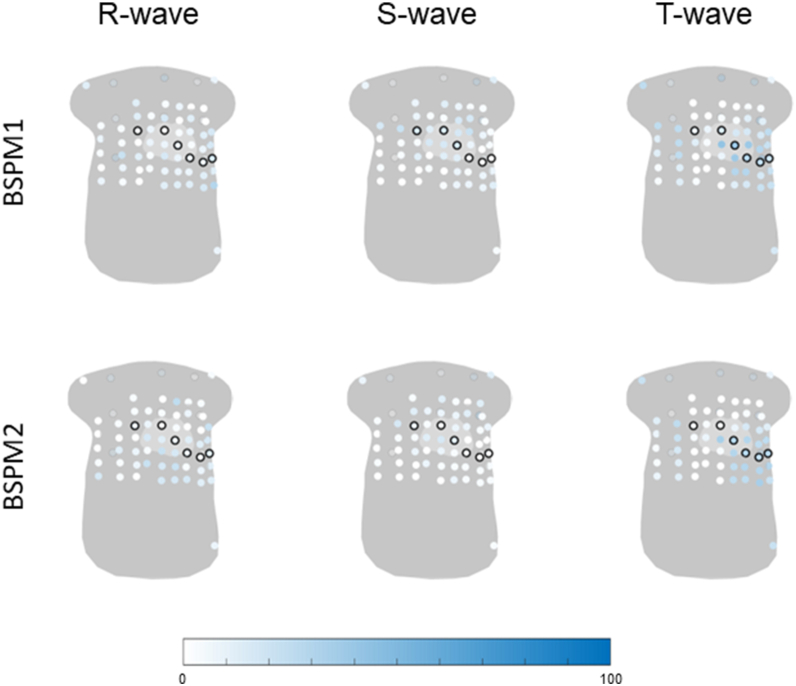
Percentage of abnormal R-, S-, or T-wave amplitude in both plakophilin-2 and phospholamban pathogenic variant carriers with and without LGE on cardiac magnetic resonance imaging. The first row shows variant carriers without LGE (n = 71), the second row carriers with RV LGE (n = 4), the third row carriers with LV LGE (n = 27), and the last row carriers with biventricular LGE (n = 10). All 67 electrodes are visualized on a general torso model, and the frequency of abnormality is visualized from *white* (0%) to *blue* (100%), where a higher color saturation indicates a higher frequency of abnormal R-, S-, and T-wave amplitudes. The standard precordial electrodes V1–V6 and the back electrodes are circled in *black*. LGE = late gadolinium enhancement; LV = left ventricle; RV = right ventricle.

Most subjects did not have LGE (47%). 10 subjects (7%) had biventricular LGE, 27 subjects (18%) had only LV LGE, and 4 subjects (3%) had only RV LGE. The R wave was less frequently abnormal in carriers without LGE (lead with the highest frequency 34%) compared with carriers with LGE (lead with the highest frequency, RV LGE 50%; LV LGE 52%; biventricular LGE 80%) (Figure 5). A similar observation was made for the S wave (lead with the highest frequency, no LGE 30%; RV LGE 25%; LV LGE 44%; biventricular LGE 80%) and T wave (lead with the highest frequency, no LGE 27%; RV LGE 50%; LV LGE 70%; biventricular LGE 100%). The location where abnormal R-, S-, and T-wave amplitudes most frequently occurred differed based on the location of LGE. In those with RV LGE, the R-wave abnormalities were mostly observed in the right-sided leads and above the standard precordial positions, whereas, in LV LGE, R-wave abnormalities were more frequent in the left inferior leads. In RV LGE, T-wave abnormalities were rarely observed in the most left-lateral leads, whereas, in those with LV LGE, this was the most common location for abnormal T-wave amplitudes. In those with biventricular LGE, locations of abnormalities were more widespread than in those with LGE in a single ventricle (Figure 5).

### CMR characteristics and arrhythmias

In *PKP2* carriers, a weak negative association was found between LVEF and the total number of leads with abnormal R-, S-, and T-wave amplitudes (Spearman’s ρ = −0.30; 95% confidence interval [CI] −0.52 to −0.27; *P* = .041), and a moderate negative association was found between RVEF and the total number of abnormal leads (Spearman’s ρ = −0.54; 95% CI −0.71 to −0.31; *P* < .001) (Supplemental Figures 1 and 2).

*PKP2* carriers with ventricular arrhythmias showed significantly more leads with abnormal R-, S-, and T-wave amplitudes than carriers without arrhythmias (9 [7;15] vs 47 [33;56] abnormal leads; *P* < .001). More abnormal leads were also observed in those with RV dyskinesia (8 [6;15] vs 29 [11;50] abnormal leads; *P* = .004) and T-wave inversions in leads V1–V3 (9 [6;15] vs 44 [29;52] abnormal leads; *P* < .001) than those without these characteristics.

In *PLN* carriers, a strong negative association was observed between LVEF and the total number of leads with abnormal R-, S-, and T-wave amplitudes (Spearman’s ρ = −0.62; 95% CI −0.75 to −0.43; *P* < .001), and a moderate negative association between RVEF and the total number of leads with abnormal amplitudes (Spearman’s ρ = −0.47; 95% CI −0.65 to −0.24; *P* < .001) (Supplemental Figures 3 and 4).

*PLN* carriers with ventricular arrhythmias showed significantly more leads with abnormal R-, S-, and T-wave amplitudes than carriers without arrhythmias (26 [7;43] vs 54 [47;61] abnormal leads; *P* = .002). More abnormal leads were also observed in carriers with LV dyskinesia (22 [7;36] vs 56 [45;63] abnormal leads; *P* < .001) and T-wave inversions in leads V4, V5, or V6 (16 [6;36] vs 56 [39;65] abnormal leads; *P* < .001) than those without these characteristics.

### Follow-up body surface potential map

In 39 carriers (Figure 2), a follow-up BSPM was performed after 4 years [3;5], among whom 23 were *PKP2*-pathogenic variant carriers (follow-up 4 [3;4] years) and 16 were *PLN*-pathogenic variant carriers (follow-up 4 [4;5] years). The heart rate at follow-up for the whole group was 61 bpm [53;67].

#### *PKP2* carriers

Of the *PKP2*-pathogenic variant carriers, 3 subjects showed disease progression during follow-up (Figure 2); they progressed from a presymptomatic stage to the electrical disease stage. Of the other subjects with a follow-up BSPM, 4 stayed in the presymptomatic disease stage, 7 remained in the electrical disease stage, and 9 had structural disease.

The number of abnormal leads was low in those in a presymptomatic disease stage who did not show any disease progression (R wave 3 [2;5]; S wave 3 [2;4]; T wave 1 [0;5] leads) and in presymptomatic variant carriers who progressed toward an electrical disease stage (R wave 1 [1;1]; S wave 2 [1;3]; T wave 1 [1;3] leads) (Table 4). A slightly higher number of abnormal leads was observed in those who remained in the electrical disease stage (R wave 3 [4;5]; S wave 2 [1;4]; T wave 3 [1;14] leads). The highest number of abnormal leads was observed in those with structural disease, where the T wave was most often abnormal (9 [7;29] abnormal leads).

**Table 4:** Number of abnormal leads for PKP2-pathogenic variant carriers and PLN-pathogenic variant carriers based on follow-up body surface potential mapping

Follow-up BSPM	All	Presymptomatic remaining presymptomatic	Presymptomatic to electrical	Electrical remaining electrical	Structural remaining structural
*PKP2*	n = 23	n = 4	n = 3	n = 7	n = 9
Abnormal R wave (n)	4 [2;7]	3 [2;5]	1 [1;1]	3 [4;5]	6 [5;13]
Abnormal S wave (n)	3 [1;6]	3 [2;4]	2 [1;3]	2 [1;4]	6 [1;9]
Abnormal T wave (n)	4 [0;14]	1 [0;5]	1 [1;3]	3 [1;14]	9 [7;29]
*PLN*	n = 16	n = 5	n = 4	n = 5	n = 2
Abnormal R wave (n)	10 [4;15]	4 [4;4]	10 [9;12]	10 [5;19]	17 [15;19]
Abnormal S wave (n)	7 [3;11]	4 [2;7]	8 [3;13]	7 [2;10]	20 [15;26]
Abnormal T wave (n)	13 [0;29]	0 [0;2]	14 [1;27]	29 [26;29]	25 [23;27]

Values are presented as median [Q1;Q3].

*BSPM* = body surface potential mapping; *PKP2* = plakophilin-2; *PLN* = phospholamban.

Among all *PKP2* carriers, no significant increase between baseline and follow-up was found in the amount of leads with abnormal R waves (baseline 5 [2;7]; follow-up 4 [2;7] leads; *P =* .300), S waves (baseline 3 [1;6]; follow-up 3 [1;6] leads; *P* = .910), and T waves (baseline 8 [1;17]; follow-up 4 [0;14] leads; *P* = .458).

**Figure 6:**
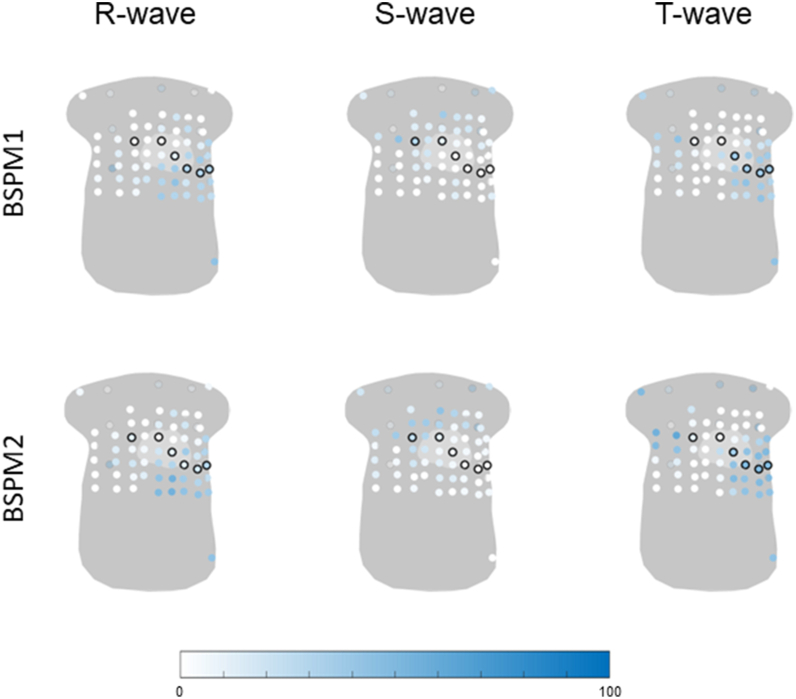
Percentage of abnormal R-, S-, or T-wave amplitude in all plakophilin-2 pathogenic variant carriers who underwent a follow-up BSPM. Their baseline BSPM is displayed in the first row, and their follow-up BSPM is displayed in the second row. All 67 electrodes are visualized on a general torso model, and the frequency of abnormality is visualized from *white* (0%) to *blue* (100%), where a higher color saturation indicates a higher frequency of abnormal R-, S-, and T-wave amplitudes. The standard precordial electrodes V1–V6 and the back electrodes are circled in *black*. BSPM = body surface potential map.

The locations of the leads and the corresponding frequency of abnormalities per R-, S-, and T-wave amplitudes for those with follow-up BSPM are shown in Figure 6 (highest frequencies, R wave 26%; S wave 30%; T wave 35%). Apart from the R wave, the locations of the leads showing the most frequent abnormalities were similar between baseline and follow-up BSPM, although the frequency of abnormalities found was higher during follow-up BSPM (maximal increase in frequency, R wave 18%; S wave 13%; T wave 13%).

#### *PLN* carriers

Of the *PLN*-pathogenic variant carriers (Figure 2), 4 subjects progressed from a presymptomatic disease stage to an electrical disease stage. Of the other subjects, 5 subjects remained in the presymptomatic stage, 5 remained in the electrical disease stage, and 2 were in a structural disease stage.

An increase in the number of abnormal leads was observed between those in a presymptomatic disease stage who remained presymptomatic at follow-up (R wave 4 [4;4]; S wave 4 [2;7]; T wave 0 [0;2] leads) and those who went from presymptomatic to an electrical disease stage at follow-up (R wave 10 [9;12]; S wave 8 [3;13]; T wave 14 [1;27] leads) (Table 4). Although the number of abnormal T waves was similar between those who remained in an electrical disease stage at follow-up (29 [26;29] leads) and those who remained in a structural disease stage (25 [23;27] leads), the amount of abnormal R and S waves was higher in those with structural disease (R wave 17 [15;19] leads; S wave 20 [15;26] leads) than electrical disease (R wave 10 [5;19] leads; S wave 7 [2;10] leads).

Among all *PLN* carriers, the number of leads with an abnormal R-wave amplitude did not differ between baseline and follow-up (baseline 6 [4;17]; follow-up 10 [4;16] leads; *P* = .277). An increase was observed in the number of abnormal S waves (baseline 4 [2;7]; follow-up 7 [3;11] leads; *P* = .002), and a nonsignificant trend in the amount of abnormal T waves (baseline 3 [0;19]; follow-up 13 [0;29] leads; *P* = .058).

**Figure 7:**
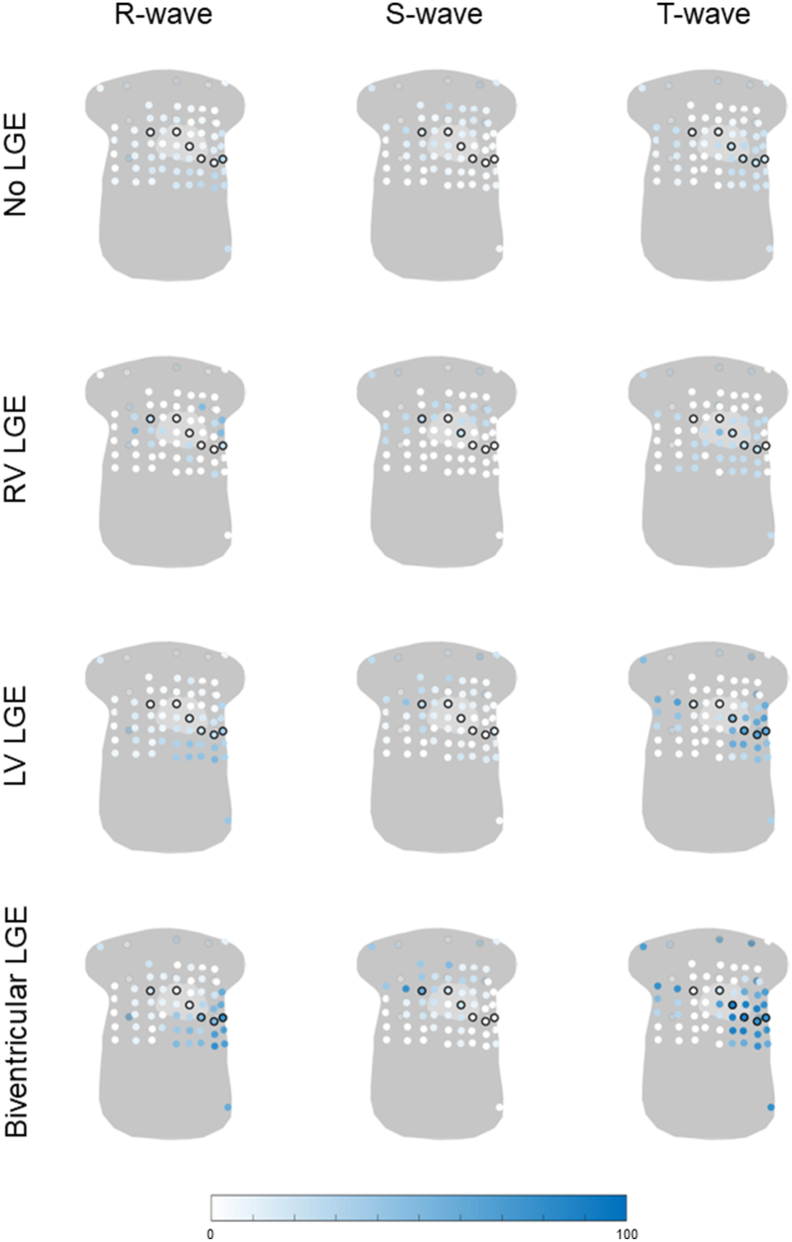
Percentage of abnormal R-, S-, or T-wave amplitude in all phospholamban pathogenic variant carriers who underwent a follow-up BSPM. Their baseline BSPM is displayed in the first row, and their follow-up BSPM is displayed in the second row. All 67 electrodes are visualized on a general torso model, and the frequency of abnormality is visualized from *white* (0%) to *blue* (100%), where a higher color saturation indicates a higher frequency of abnormal R-, S-, and T-wave amplitudes. The standard precordial electrodes V1–V6 and the back electrodes are circled in *black*. BSPM = body surface potential map.

The locations of the leads and the corresponding frequency of abnormalities per R-, S-, and T-wave amplitudes for those with follow-up BSPM are shown in Figure 7 (highest frequencies, R wave 56%; S wave 44%; T wave 63%). The leads showing abnormalities overlapped between baseline and follow-up BSPM for all 3 waveforms, with an increased frequency of abnormalities during follow-up BSPM (maximal increase in abnormal frequency, R wave 25%; S wave 25%; T wave 31%).

## Discussion

This study applied BSPM to assess cardiac activation and repolarization in the largest cohort to date of *PKP2* and *PLN* cardiomyopathy, both associated with the rare disease ACM. Abnormalities in ECG signals were observed both within and beyond the 12-lead configuration in pathogenic variant carriers associated with ACM. Notably, these abnormalities were detectable even in presymptomatic variant carriers. Findings of follow-up BSPM suggest that these abnormalities are associated with disease progression.

### Disease stages

Abnormalities in R-, S-, and T-wave amplitudes were observed across all disease stages in both *PKP2*- and *PLN*-pathogenic variant carriers, with the highest frequency occurring in the structural stage. Notably, even presymptomatic individuals showed abnormalities compared with control subjects. This was more apparent in *PLN*-variant carriers than *PKP2*-variant carriers at both baseline and follow-up. Therefore, the detection of R-, S-, and T-wave amplitudes by BSPM might be a sensitive method to detect (early) electrical abnormalities in pathogenic variant carriers at risk of cardiomyopathy.

The locations of electrodes showing the most frequent abnormalities were often located outside the 12-lead configuration (below the left-sided precordial leads [V3–V6] and beyond the right-sided precordial leads [V1–V2]). In particular, in presymptomatic *PLN* carriers, the leads that showed abnormalities most frequently were farther away from the standard precordial positions. As the disease progressed, the distribution of electrodes with abnormal amplitudes expanded toward more central regions of the torso, including V1–V6. These findings highlight the potential value of a more extensive lead system than the standard 12-lead ECG, given that BSPM may detect subtle abnormalities in presymptomatic carriers that are not captured by conventional ECG.

Previous smaller ECG/BSPM studies on *PLN*^19^ and desmosomal variants^10^, ^11^, ^12^^,^^20^ associated with ACM already showed that, in a subset of presymptomatic carriers, electrical abnormalities were found. Consistent with our findings, the greatest abnormalities were found in leads outside the 12-lead configuration. This study now confirms these findings in a larger cohort and across multiple genetic backgrounds, while using only R-, S-, and T-wave amplitudes.

### Variant-specific findings

Abnormalities in amplitudes were more frequently observed in *PLN*-pathogenic variant carriers than *PKP2*-pathogenic variant carriers. Given that low-voltage ECGs are described in *PLN*,^5^^,^^19^ it is expected that the amplitude-based method used in this study is more sensitive for this specific group of variant carriers.

Interestingly, although *PKP2* is typically described as an RV disease and *PLN* more as a biventricular disease,^6^ the locations of abnormal leads were strikingly similar between the 2 groups of variant carriers, particularly for T-wave abnormalities. Even though R-wave abnormalities were more apparent in the left lower leads in *PLN* carriers, in *PKP2* carriers, the right-sided and upper leads were also frequently affected. The S wave seems to be more affected in right-sided leads in *PLN* carriers, whereas in *PKP2* carriers the central/left upper leads are more affected. The differences in locations of abnormal R and S waves on the body surface between *PKP2* and *PLN* genetic variants may not be solely attributed to the variant-specific locations of disease manifestation. They may also reflect variant-specific electrophysiological characteristics. In *PLN*, widespread microvoltages^5^^,^^19^ can lead to diffuse abnormalities in the R, S, and T waves. In contrast, *PKP2*-associated abnormalities are more likely to be the result of delayed conduction^21^^,^^22^ and thereby altered activation sequences even when ECG amplitudes are (largely) preserved.^10^

### Disease progression

Previous research on follow-up BSPM in healthy subjects^23^ investigated amplitude changes caused by external factors such as respiration and repeated electrode placement, with the greatest variation observed near the standard precordial positions V1–V6. In contrast, the current study in pathogenic variant carriers revealed abnormal amplitudes primarily in electrodes located in the lower left and right regions of the torso, suggesting a pattern caused by disease instead of normal physiological variability.

The current study demonstrated the extent of electrical abnormalities through different time points in pathogenic variant carriers. Follow-up BSPM showed that leads identified as abnormal at baseline also showed abnormalities at follow-up BSPM. Besides leads becoming more frequently abnormal, neighboring leads also become more abnormal than baseline BSPM. This suggests that, as the disease progresses, although this was not always reflected by a transition to a more advanced disease stage, the abnormal leads at baseline may already reflect early electrical manifestations of the disease. Given that ventricular arrhythmias may be the earliest sign of disease manifestation, it is of great importance to find early signs of (electrical) abnormalities that may indicate a greater risk of life-threatening events. The abnormalities in presymptomatic carriers detected by BSPM may offer an insight into this process, although further follow-up is necessary to determine the predictive value of these findings.

Median follow-up duration was 4 years and was similar between *PKP2* and *PLN* carriers. Nevertheless, a higher increase in abnormal leads between baseline and follow-up was observed in *PLN* carriers than *PKP2* carriers. In *PKP2*, only 3 of 23 (13%) showed disease progression by transitioning from a presymptomatic to electrical stage, whereas in *PLN* 4 of 16 (25%) progressed toward a new disease stage. This discrepancy, as well as the higher number of abnormal leads that occur in the general *PLN* population than *PKP2*, might explain the differences in follow-up findings between the 2 groups. Earlier research on presymptomatic *PKP2*/*PLN* carriers also suggests that progression toward electrical disease (determined by the development of ventricular arrhythmias during follow-up) occurs more rapidly in *PLN*^18^ than in *PKP2*.^24^

In *PLN* carriers, an increase in abnormal leads was observed in presymptomatic carriers who progressed toward an electrical disease stage, whereas, in *PKP2* carriers, this was not observed. However, the number of abnormal leads at baseline BSPM was already similar between *PKP2* carriers in the presymptomatic and the electrical stage, suggesting that progression to an electrical disease stage does not increase the effect on R-, S-, and T-wave amplitudes in this specific gene variant. Therefore, analysis of R-, S-, and T-wave amplitudes of BSPM might be more useful in *PLN* carriers than *PKP2* carriers, given that it more accurately reflects disease progression. These results further underline that genotype-specific (ECG) criteria are likely more beneficial in determining disease expression in the spectrum of ACM.

### Relation to clinical features

A higher amount of abnormal R-, S-, and T-wave amplitudes was observed in carriers with ventricular arrhythmias. The same metrics as used in our study, T-wave inversions and low amplitudes, have been shown to be predictors of sustained ventricular arrhythmias in ARVC^25^ and *PLN* cardiomyopathy.^26^ Given that abnormal amplitudes were more frequently observed outside the precordial lead positions V1–V6, abnormal leads identified by BSPM may likewise signal an elevated risk of ventricular arrhythmias, which could be overlooked by a conventional ECG analysis.

The findings of this study also suggest that electrical abnormalities detected by BSPM reflect underlying structural abnormalities, given that abnormal amplitudes were more often found in those with LV/RV dyskinesia, and locations of LGE corresponded with different torso regions that demonstrated abnormal amplitudes.

### Methodological considerations

Different classifications for the disease stages were used between *PKP2* and *PLN* in this study, given that we used TFC-based criteria for *PKP2* carriers and more LV-oriented criteria in *PLN* carriers. In *PKP2* carriers, the number of subjects in the presymptomatic disease stage (17 [32%]) was similar to those with TFC of ≤2 (18 [34%]). This was not the case in *PLN* carriers where, based on the TFC, the presymptomatic group would be much larger (44 [75%]) than when using an LV-oriented classification system (32 [54%]). This underlines that the TFC might not be appropriate to determine the presence of disease expression in *PLN* carriers.

For this study, we only investigated R-, S-, and T-wave amplitudes and did not look into other segments of the ECG, such as the ST segment, or variant-specific ECG abnormalities, such as prolonged terminal activation duration and T-wave negativity. However, the use of normal ranges per amplitude would also register negative T waves in abnormal locations, so these abnormalities should also have been detected using this amplitude-based method.

### Limitations

Limitations of this study include the inclusion of subjects referred for CMR/CT in our control cohort, given that they were referred owing to a suspicion or exclusion of cardiac disease. However, more than half of the control group consisted of healthy subjects/athletes without cardiac symptoms, and those with cardiac symptoms all had normal echocardiography and/or CMR, and no cause for their symptoms was found. Athletes are known to have slightly different volumetrics than the normal population^27^; this is also reflected in the relatively large RV outflow tract diameter of our control population. However, including these subjects as controls increased the sample size and broadened the age range and variability in ECG amplitudes, which is likely beneficial for defining normal ranges. Furthermore, the sex distribution was different between controls and *PLN* carriers, given that more female *PLN* carriers were included than in the control population, which might have influenced the results. Nonetheless, sufficient females were included (39%) to also have a well-defined female normal range.

A major limitation of this study is the small sample size during follow-up, especially within the subanalysis stratified by disease stage. As a result, the findings should be interpreted with caution, considering the low numbers in each subgroup. Owing to these small numbers, statistical significance testing was not performed between the disease stages at follow-up. A bigger follow-up cohort would have improved the interpretability of these results. However, the findings of the follow-up BSPM are consistent with the findings from baseline analysis and are therefore likely representative.

We did not correct for heart rate differences, which might have an important influence on the R-, S-, and T-wave amplitudes.^23^ However, the range in heart rates in our control group was comparable with that of the genetic variant carriers, as well as between baseline BSPM and follow-up BSPM. Therefore, it is expected that correction for heart rate differences is not needed.

Finally, BSPM is currently not used as a clinical diagnostic but as a research tool. This limits the current clinical relevance of these findings.

### Future perspective

As shown in this study, BSPM might be of added value in the early detection of disease in presymptomatic variant carriers, especially in *PLN* cardiomyopathy. By incorporating ECG imaging—an approach that combines BSPM with CMR or CT to noninvasively estimate activation and repolarization times on the cardiac surface—future studies should provide deeper insights into the electrophysiological mechanisms underlying the observations in the present study. Better understanding and monitoring of changes in depolarization and repolarization will be of value not only in early diagnosis but also in the treatment of patients prone to ventricular arrhythmias.

Follow-up data should be further collected over time to determine the association of BSPM abnormalities—in particular in presymptomatic pathogenic variant carriers—with the development of ventricular arrhythmias and the need for cardioverter defibrillator implantation to assess the clinical consequences of these abnormalities. A larger cohort will be necessary, given that the subgroups per disease stage were still relatively small. Moreover, although this study focused on carriers of *PKP2* and *PLN* genetic variants, future studies should also look into other genetic variants such as desmoplakin and desmoglein-2.

## Conclusion

This study demonstrated abnormal R-, S-, and T-wave amplitudes within and beyond the 12-lead configuration in *PKP2* and *PLN* cardiomyopathy. Abnormalities were already present in presymptomatic variant carriers, typically occurring outside standard precordial lead positions. Findings of follow-up BSPM suggest that these abnormalities are associated with disease progression. With more prominent amplitude changes in *PLN* carriers than *PKP2* carriers, analysis of R-, S-, and T-wave amplitudes in BSPM might be more useful in *PLN* carriers than *PKP2* carriers.
