# Supplementary material for: Body surface potential mapping of ventricular depolarization and repolarization in phospholamban and plakophilin-2 cardiomyopathy
Source: Heart Rhythm O2. 2025 Oct 17;7(1):130–42. doi: 10.1016/j.hroo.2025.09.027 (PMC12902137; doi:10.1016/j.hroo.2025.09.027)
Supplement: Supplemental Figures 1-4 [file mmc1.docx]

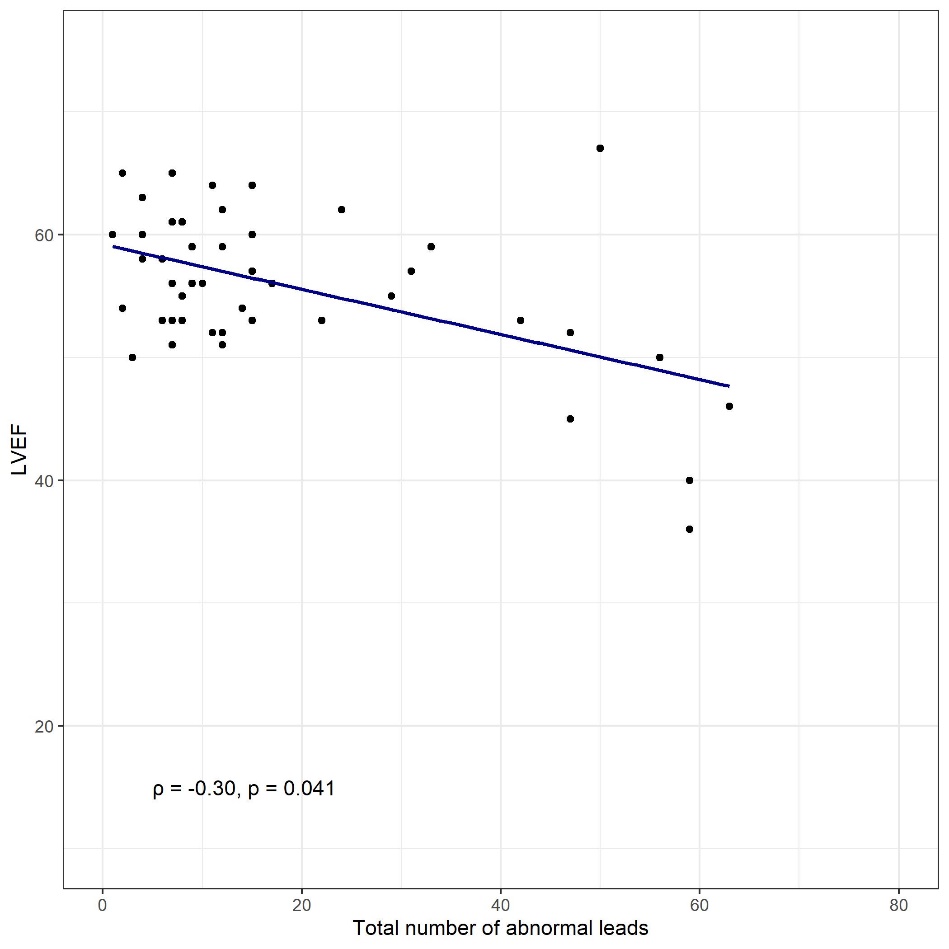


**Supplemental Figure 1**. Correlation plot between the total number of abnormal leads due to an abnormal amplitude and the left ventricular ejection fraction (LVEF) for Plakophilin-2 pathogenic variant carriers.


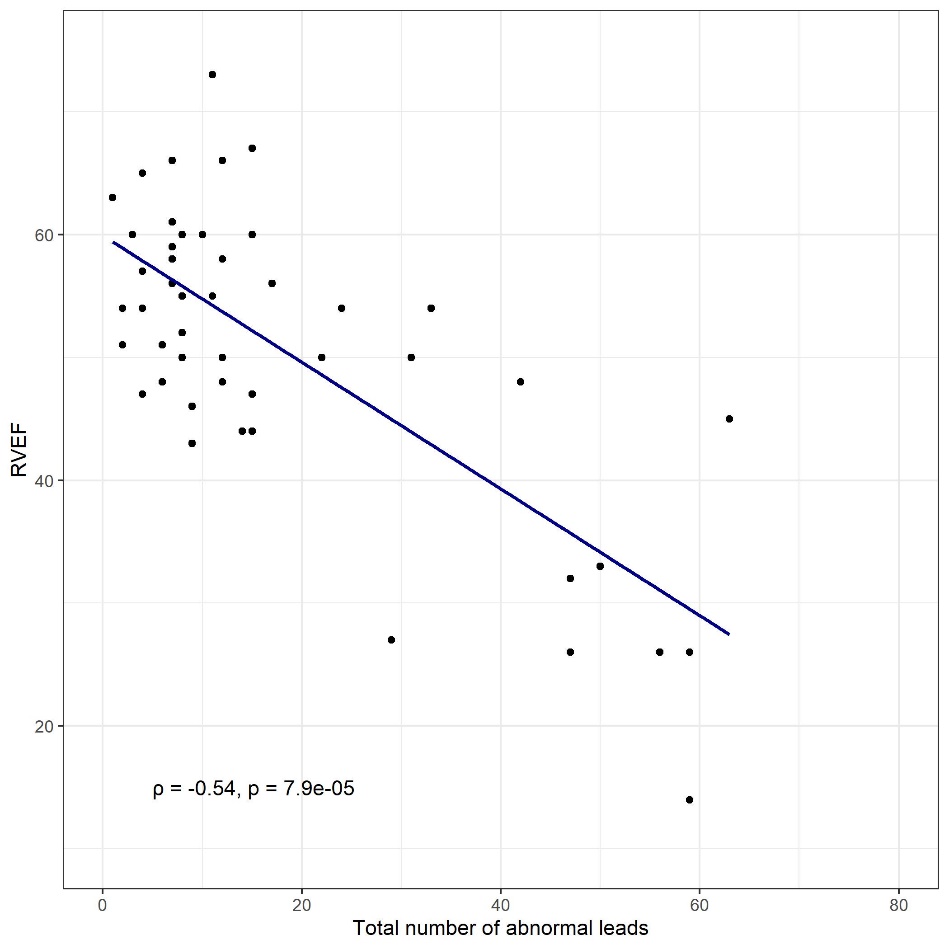


**Supplemental Figure 2**. Correlation plot between the total number of abnormal leads due to an abnormal amplitude and the right ventricular ejection fraction (RVEF) for Plakophilin-2 pathogenic variant carriers.


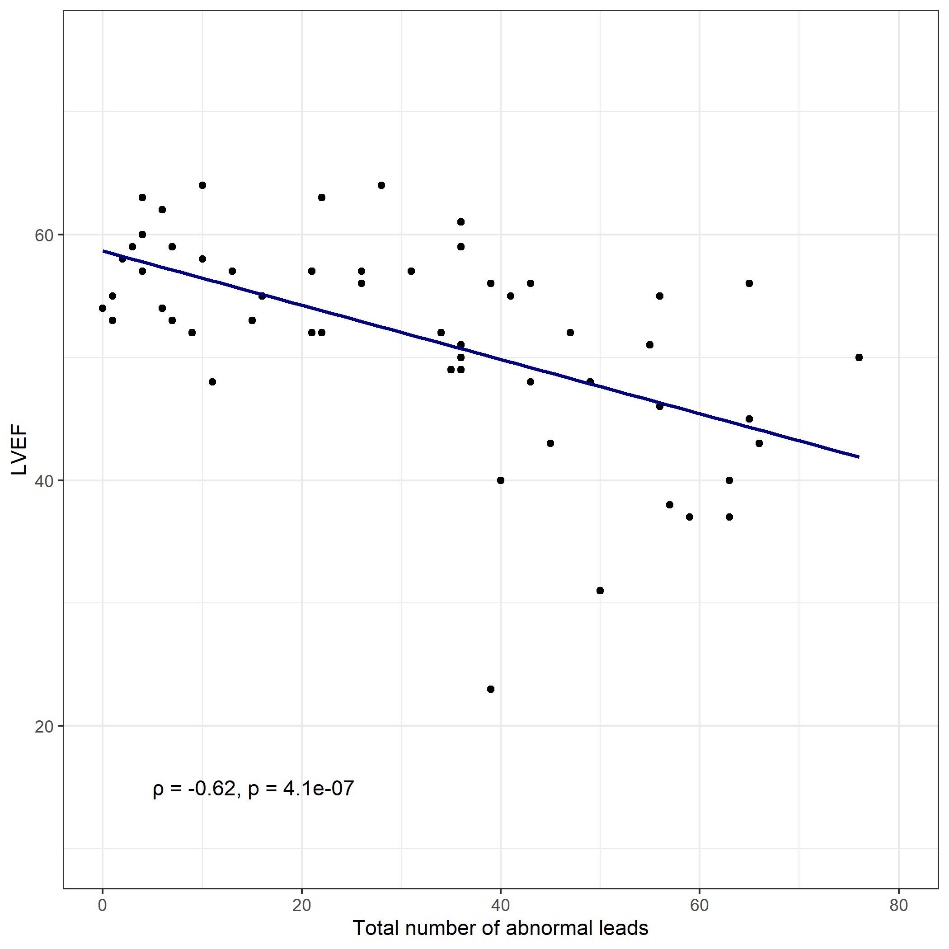


**Supplemental Figure 3**. Correlation plot between the total number of abnormal leads due to an abnormal amplitude and the left ventricular ejection fraction (LVEF) for Phospholamban pathogenic variant carriers.


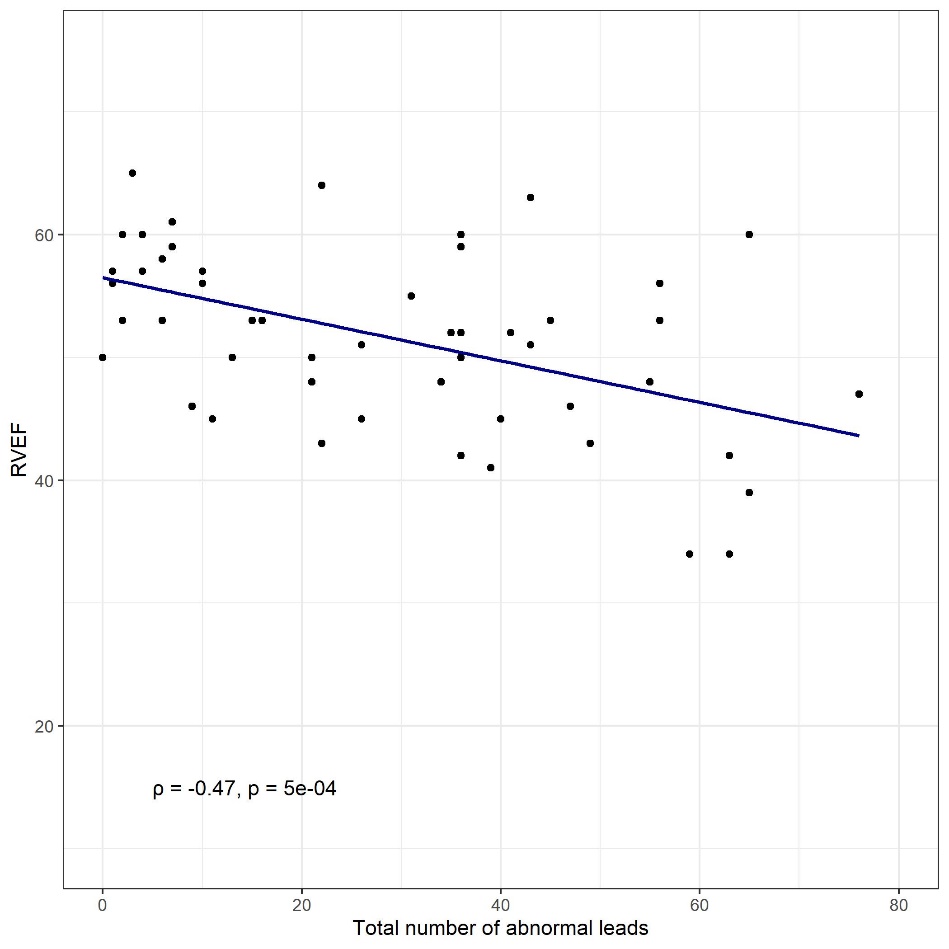


**Supplemental Figure 4**. Correlation plot between the total number of abnormal leads due to an abnormal amplitude and the right ventricular ejection fraction (RVEF) for Phospholamban pathogenic variant carriers.
